# Impact of log parsing on deep learning-based anomaly detection

**DOI:** 10.1007/s10664-024-10533-w

**Published:** 2024-08-17

**Authors:** Zanis Ali Khan, Donghwan Shin, Domenico Bianculli, Lionel C. Briand

**Affiliations:** 1 University of Luxembourg, Esch-sur-Alzette, Luxembourg; 2 University of Sheffield, Sheffield, United Kingdom; 3 University of Ottawa, Ottawa, Canada; 4 The Lero SFI Centre for Software Research, Limerick, Ireland; 5 University of Limerick, Limerick, Ireland

**Keywords:** Logs, Log parsing, Template identification, Anomaly detection

## Abstract

Software systems log massive amounts of data, recording important runtime information. Such logs are used, for example, for log-based anomaly detection, which aims to automatically detect abnormal behaviors of the system under analysis by processing the information recorded in its logs. Many log-based anomaly detection techniques based on deep learning models include a pre-processing step called log parsing. However, understanding the impact of log parsing on the accuracy of anomaly detection techniques has received surprisingly little attention so far. Investigating what are the key properties log parsing techniques should ideally have to help anomaly detection is therefore warranted. In this paper, we report on a comprehensive empirical study on the impact of log parsing on anomaly detection accuracy, using 13 log parsing techniques, seven anomly detection techniques (five based on deep learning and two based on traditional machine learning) on three publicly available log datasets. Our empirical results show that, despite what is widely assumed, there is no strong correlation between log parsing accuracy and anomaly detection accuracy, regardless of the metric used for measuring log parsing accuracy. Moreover, we experimentally confirm existing theoretical results showing that it is a property that we refer to as distinguishability in log parsing results—as opposed to their accuracy—that plays an essential role in achieving accurate anomaly detection.

## Introduction

Software system execution logs provide valuable information about the runtime behavior of the system, which is essential for monitoring and troubleshooting. Among many log analysis approaches, *log-based anomaly detection* has been actively studied to automatically detect abnormal behaviors of the system under analysis by processing the information recorded in logs (He et al. 2021). Recently, anomaly detection techniques based on Deep Learning (DL) models, such as Long Short-Term Memory (LSTM) (Du et al. 2017; Meng et al. 2019; Zhang et al. 2019) and Convolutional Neural Networks (CNNs) (Lu et al. 2018), have shown promising results.

One common aspect of most anomaly detection techniques is having a pre-processing step called *log parsing* (also known as log template identification). This step is needed because anomaly detection techniques require structured logs to automatically process them, whereas input logs are often free-formed or semi-structured, as generated by logging statements (e.g., printf() and logger.info()) in the source code. Many log parsing techniques have also been developed to automatically convert unstructured input logs into structured logs (Zhu et al. 2019).

The frequent combination of log parsing and anomaly detection clearly implies the importance of the former for the latter. Nevertheless, assessing in a systematic way the impact of log parsing on anomaly detection has received surprisingly little attention so far. Only recently, Shin et al. (2021) investigated what *ideal* log parsing results are in terms of accurate anomaly detection, but purely from a theoretical standpoint. Le and Zhang (2022) empirically showed that different log parsing techniques, among other potential factors, can significantly affect anomaly detection accuracy, but the accuracy of log parsing results was not adequately measured, and the correlation between log parsing accuracy and anomaly detection accuracy was not reported. Fu et al. (2023) attempted to address the issue by evaluating log parsing and anomaly detection accuracy. However, they relied on a single log parsing accuracy metric (Khan et al. 2022), and the log parsing results used to evaluate anomaly detection techniques were based on less than 1% of all logs used, which limits the validity of the findings.

To systematically investigate the impact of log parsing on anomaly detection while addressing the issues of the aforementioned studies, this paper reports on an empirical study, in which we performed a comprehensive evaluation using 13 log parsing techniques, seven anomaly detection techniques—five based on deep learning and two based on traditional machine learning—on three publicly available log datasets. We considered all three log parsing accuracy metrics (i.e., grouping accuracy (Zhu et al. 2019), parsing accuracy (Dai et al. 2020), and template accuracy (Khan et al. 2022)) proposed in the literature.

Against all assumptions, our results show that there is no strong correlation between log parsing accuracy and anomaly detection accuracy, regardless of the metric used for measuring log parsing accuracy. In other words, accurate log parsing results do not necessarily increase anomaly detection accuracy. To better understand the phenomenon at play, we investigated another property of log parsing, *distinguishability*, a concept proposed by Shin et al. (2021) that was theoretically shown to relate to anomaly detection accuracy. Our empirical results confirm that, as far as anomaly detection is concerned, distinguishability in log parsing results is the property that really matters and should be the key target of log parsing.

In summary, the main contributions of this paper are:

- the systematic and comprehensive evaluation of the impact of log parsing on anomaly detection;
- the investigation of the impact of the distinguishability of log parsing results on anomaly detection.

The rest of the paper is organized as follows. Section 2 provides basic information used throughout the paper, including the definitions of logs, messages, and templates, as well as an overview of log parsing and anomaly detection. Section 3 motivates our study and introduces the research questions. Section 4 describes the experimental design, including the log datasets, log parsing techniques, and anomaly detection techniques used in the experiments. Section 5 presents the experimental results. Section 6 discusses the practical implications, derived from the results, for the application of log parsing in the context of anomaly detection. Section 7 surveys the related work. Section 8 concludes the paper and provides directions for future work.

## Background

In this section, we provide an overview of the main concepts that will be used throughout the paper. We first introduce the definitions of logs, messages, and log templates (§ 2.1). We then explain the concept of log parsing (also known as log template identification) and illustrate different log parsing accuracy metrics proposed in the literature (§ 2.2). We discuss log-based anomaly detection and the corresponding accuracy metrics in § 2.3. Finally, we summarize the recent theoretical results on ideal log parsing for accurate anomaly detection, introducing the concept of *distinguishability* for log parsing results (§ 2.4).

### Logs, messages, and templates

A *log* is a sequence of log entries^1^. A *log entry* contains various information about the event being logged, including a timestamp, a logging level (e.g., INFO, DEBUG), and a log message. A *log message* can be further decomposed into fixed and variable parts since it is generated by executing a logging statement that can have both fixed (hard-coded) strings and program variables in the source code. For example, the execution of the logging statement “logger.info("Deleting block " + blkID + " file " + fileName)” when the program variables blkID and fileName evaluate to blk-1781 and /hadoop/dfs, respectively, will generate a log entry “11:22:33 INFO Deleting block blk-1718 file /hadoop/dfs” where the log message “Deleting block blk-1718 file /hadoop/dfs” can be decomposed into the fixed parts (i.e., “Deleting block” and “file”) and the variable parts (i.e., “blk-1718” and “/hadoop/dfs”). A *(log message) template* masks the various elements of each variable part with a special character “<*>”; this representation is widely used in log-based analyses (e.g., log parsing (He et al. 2017; Jiang et al. 2008), anomaly detection (Zhang et al. 2019; Du et al. 2017), and log-based testing (Elyasov 2012; Jeong et al. 2020)) when it is important to focus on the event types captured by a log message.

For instance, the template corresponding to the example log message “Deleting block blk-1178 file /hadoop/dfs” is “Deleting block<*> file<*>”.

### Log parsing (Log Template Identification)

Although software execution logs contain valuable information about the run-time behavior of the software system under analysis, they cannot be directly processed by log-based analysis techniques that require structured input logs (containing templates) instead of free-formed log messages. Extracting log templates from log messages is straightforward when the source code with the corresponding logging statements is available. However, often the source code is unavailable, for example, due to the usage of 3rd-party, proprietary components. This leads to the problem of log parsing (log template identification): *How can we identify the log templates of log messages without accessing the source code?*

To address this problem, many automated log-parsing approaches, which take as input log messages and identify their log templates using different heuristics, have been proposed in the literature (e.g., AEL (Jiang et al. 2008), Drain (He et al. 2017), IPLoM (Makanju et al. 2009), LenMa (Shima 2016), LFA (Nagappan and Vouk 2010), LogCluster (Vaarandi and Pihelgas 2015), LogMine (Hamooni et al. 2016), Logram (Dai et al. 2020), LogSig (Tang et al. 2011), MoLFI (Messaoudi et al. 2018), SHISO (Mizutani 2013), SLCT (Vaarandi 2003), and Spell (Du and Li 2016)).

Three different accuracy metrics have been proposed to evaluate the accuracy of log parsing approaches: *Grouping Accuracy* (GA) Zhu et al. (2019), *Parsing Accuracy* (PA) Dai et al. (2020), and *Template Accuracy* (TA) Khan et al. (2022).

Zhu et al. (2019) observed that log parsing can be considered as a clustering process where log messages with the same template are clustered into the same group. Based on this idea, they proposed the GA metric to assess if log messages are correctly grouped. Specifically, GA is defined as the ratio of log messages *correctly parsed* by the log parsing approach under evaluation over the total number of log messages, where a log message is correctly parsed when its log message group is the same as the ground truth (i.e., a group generated by oracle templates).

Dai et al. (2020) later proposed PA, to address the issue that GA only considers message groups, not the equivalence between the templates identified by the log parsing approach under evaluation and the oracle templates. Although having correctly grouped messages would be enough in some cases (e.g., detecting anomalies based on the sequence of template IDs without considering the content of the templates (Du et al. 2017)), correctly identified templates (i.e., templates identical to the corresponding oracle ones) matter when the fixed parts of templates are used (e.g., detecting anomalies based on the semantic information in the templates (Zhang et al. 2019)). To this end, PA replaces the definition of a correctly parsed log message in GA as follows: a log message is *correctly parsed* when its identified template is identical to the oracle template.

Khan et al. (2022) recently proposed the TA metric, since both GA and PA are defined based on the number of correctly parsed log messages and, therefore, can be misleading, especially when there are many repeated messages (e.g., heartbeat messages). Specifically, they introduced Precision-TA (PTA) and Recall-TA (RTA), where PTA is defined as the number of correctly identified templates over the total number of identified templates and RTA is defined as the number of correctly identified templates over the total number of oracle templates. Moreover, FTA (short for “F1-measure TA”) is the harmonic mean of PTA and RTA.

### Anomaly detection

(Log-based) anomaly detection is a technique that aims to identify anomalous patterns, recorded in input logs, that do not conform to the expected behaviors of the system under analysis (He et al. 2021). It takes as input a sequence of log templates and determines whether the given sequence represents a normal behavior of the system or not.

With the recent advances in Deep Learning (DL), many anomaly detection approaches, which leverage DL models to learn various aspects of log template sequences of normal and abnormal behaviors and classify them, have been proposed in the literature; for example, DeepLog (Du et al. 2017), LogAnomaly (Meng et al. 2019), and LogRobust (Zhang et al. 2019) are based on Long Short-Term Memory based (LSTM), CNN (Lu et al. 2018) is based on Convolutional Neural Network, and PLELog (Yang et al. 2021) is based on Gated recurrent units (GRUs).

To assess the accuracy of anomaly detection approaches, it is common practice to use standard metrics from the information retrieval domain, such as *Precision*, *Recall*, and *F1-Score*. These metrics are defined as follows: $\textit{Precision} = \frac{\textit{TP}}{\textit{TP} + \textit{FP}}$, $\textit{Recall} = \frac{\textit{TP}}{\textit{TP} + \textit{FN}}$, and $\textit{F1-score} = \frac{2\times \textit{Precision} \times \textit{Recall}}{\textit{Precision} + \textit{Recall}}$ where *TP* (True Positive) is the number of abnormal logs correctly identified by the model, *FP* (False Positive) is the number of normal logs incorrectly identified as anomalies by the model, and *FN* (False Negative) is the number of abnormal logs incorrectly identified as normal.

### Ideal log parsing results for accurate anomaly detection

Given the dependency of anomaly detection on log parsing, Shin et al. (2021) presented a theoretical analysis on *ideal* log parsing results for accurate anomaly detection. The idea behind the analysis is that log parsing can be regarded as the abstraction of log messages, where some tokens in the messages are converted to variable parts. Then, if normal and abnormal logs are over-abstracted by log parsing so that they are *indistinguishable* from each other, it is clear that anomaly detection, which takes as input the parsed logs (i.e., abstracted logs, sequences of templates), cannot distinguish normal from abnormal logs. Based on this idea, they formally defined the concept of *distinguishability* as a property of log parsing results and showed that it is an essential condition for ideal log parsing results.

Specifically, let *M* be a set of log messages and *L* be a set of logs where a log $$l\in L$$ is a sequence of log messages $$\langle m_1, m_2 , \dots , m_n \rangle $$. Also, let $$L_n \subseteq L$$ be a set of normal logs and $$L_a \subseteq L$$ be a set of abnormal logs such that $$L_n \cap L_a = \emptyset $$ and $$L_n \cup L_a = L$$. Given *M* and a set of templates (i.e., log parsing results) *T*, an abstraction function $$\tau : M \rightarrow T$$ that represents a generic log parsing approach can be defined. Based on $$\tau $$, an abstraction of a log $$l = \langle m_1, m_2 , \dots , m_n \rangle $$ can be defined as $$\tau ^*(l) = \langle \tau (m_1), \tau (m_2) , \dots , \tau (m_n) \rangle $$. Similarly, an abstraction of a set of logs *L* can be defined as $$\tau ^{**}(L) = \{\tau ^*(l) \mid l \in L\}$$. Notice that $$\tau ^{**}(L)$$ represents a log parsing result for a set of logs *L*.

The notion of distinguishability can be defined as follows: $$\tau $$ *distinguishes* $$L_n$$ and $$L_a$$ if and only if $$\tau ^{**}(L_n) \cap \tau ^{**}(L_a) = \emptyset $$. In other words, a log parsing approach distinguishes between normal and abnormal logs if and only if they are still distinguishable after log parsing. When $$\tau $$ distinguishes $$L_n$$ and $$L_a$$, $$\tau ^{**}(L)$$ for $$L = L_n \cup L_a$$ is called *d-maintaining*, meaning that the distinguishability between $$L_n$$ and $$L_a$$ is *maintained* in the log parsing result.

## Motivation

As discussed in Section 2, log parsing converts unstructured logs into structured ones, which can then be processed by log-based analysis techniques like anomaly detection. It is quite natural to speculate that log parsing results can affect anomaly detection results. Intuitively, the research literature has assumed that inaccurate log parsing results leads to inaccurate anomaly detection results. However, this hypothesis has not been fully investigated in the literature, except for one empirical study (Le and Zhang 2022) and one analytical investigation (Shin et al. 2021).

Le and Zhang (2022) recently presented an empirical work investigating several aspects that can impact Deep Learning (DL)-based anomaly detection approaches, such as training data selection, data grouping, class distribution, data noise, and early detection ability. One of their experiments considering data noise assessed the impact of noise deriving from log parsing results. Specifically, they used four log parsing techniques (Drain (He et al. 2017), Spell (Du and Li 2016), AEL (Jiang et al. 2008), and IPLoM (Makanju et al. 2009)) to generate log parsing results for two log datasets (BGL (Oliner and Stearley 2007) and Spirit (Oliner and Stearley 2007)). Then, for each log dataset, they used the different log parsing results as input of five anomaly detection approaches (DeepLog (Du et al. 2017), LogAnomaly (Meng et al. 2019), PLELog (Yang et al. 2021), LogRobust (Zhang et al. 2019), and CNN (Lu et al. 2018)), and measured the accuracy of the latter. Their experimental results showed that log parsing approaches highly influence the accuracy of anomaly detection; for example, the F1-Score of DeepLog on Spirit logs (Oliner and Stearley 2007) decreases from 0.755 to 0.609 when Drain is used instead of IPLoM for log parsing.

Although this is the first clear evidence showing the impact of log parsing results on anomaly detection accuracy, the scope of the underlying study is limited. For example, it simply uses different log parsing results (produced by different tools) without quantitatively assessing the accuracy of the log parsing tools; therefore, the relationship between log parsing accuracy and anomaly detection accuracy remains unclear. To this end, we define our first research question as follows: ***RQ1 - To which extent does the accuracy of log parsing affect the accuracy of anomaly detection?***

As summarized in Section 2.4, Shin et al. (2021) recently proposed a theoretical framework determining the ideal log parsing results for anomaly detection by introducing the concept of “distinguishability” for log parsing results. It is argued that, rather than accuracy as previously assumed, what really matters is the extent to which log parsing results are distinguishable. However, to the best of our knowledge, there is no empirical work assessing quantitatively distinguishability in log parsing results and its impact on anomaly detection accuracy. Therefore, we define our second research question as follows: ***RQ2 - How does the accuracy of anomaly detection vary with distinguishability of log parsing results?***

Answering the above questions will have a significant impact on both research and industry in the field of log-based anomaly detection. For example, if the answer to the first question is that, regardless of the log parsing accuracy metrics, there is no relationship between log parsing accuracy and anomaly detection accuracy, then it means that there is no need to use the existing accuracy metrics to evaluate log parsing results for anomaly detection. This would completely change the way log parsing tools are evaluated. Similarly, if the answer to the second question is that the distinguishability of log parsing results indeed affects anomaly detection, as expected from the recent theoretical analysis (Shin et al. 2021), then this must be the focus of log parsing evaluations. As a result, our answers will provide essential insights on better assessing the quality of log parsing techniques for more accurate anomaly detection.

## Experimental design

All experiments presented in this paper were carried out using the HPC facilities of the University of Luxembourg (see https://hpc.uni.lu). Specifically, we used Dual Intel Xeon Skylake CPU (8 cores) and 64GB RAM for running individual log parsing and anomaly detection techniques.

### Datasets

To answer the research questions introduced in Section 3, we used publicly available datasets based on the LogHub benchmark (He et al. 2020), which contains a large collection of log messages from various types of systems including operating systems (Linux, Windows, and Mac), distributed systems (BGL, Hadoop, HDFS, Thunderbird, and OpenStack), standalone programs (Proxifier and Zookeeper), and mobile systems (Android). The benchmark has been widely used in various studies focused on log parsing (Khan et al. 2022; Zhu et al. 2019; Dai et al. 2020) and anomaly detection (Le and Zhang 2022; Fu et al. 2023).

Among the benchmark datasets, we selected HDFS, Hadoop, and OpenStack datasets because of the following reasons: (1) they have labels for normal and abnormal logs to be used for assessing the accuracy of anomaly detection techniques *and* (2) the source code of the exact program version used to generate the logs is publicly available; this allows us to extract correct oracle templates (i.e., ground truth templates) for each log message. The oracle templates are especially important in our study as we need to carefully assess both log parsing accuracy and anomaly detection accuracy. Although the benchmark provides some oracle templates for all log datasets, they are *manually generated* (without accessing the source code) and cover *only* 2K log messages randomly sampled for each dataset. As discussed by Khan et al. (2022), those manually generated oracle templates are *error-prone*; therefore, we used the logging statements in the source code to extract correct oracle templates. Table 1 shows all the log datasets in the LogHub benchmark and whether they meet each of the above-mentioned criteria; the rows highlighted in gray meet both criteria.

During our preliminary evaluation, we found an issue with HDFS. The original HDFS logs were too large (11.2M log messages) to be processed by the slowest anomaly detection technique (i.e., LogAnomaly (Meng et al. 2019)) when setting a two-day timeout. Due to the large number of experiments we needed to conduct (i.e., all combinations of log parsing and anomaly detection techniques with additional repeats for distinguishable and indistinguishable log parsing results, see § 4.4 and § 4.5), we decided to reduce the log dataset size. As we found that the slowest log parsing technique (i.e., LogAnomaly) could process up to $n=300K$ messages within 2 h, we randomly and iteratively removed logs (i.e., sequences of log messages) from the HDFS dataset to reduce it until the total number of remaining messages was less than 300K. Notice that each HDFS log is a sequence of log messages having the same block ID, representing either a normal or abnormal sequence of events. To preserve individual (normal or abnormal) sequences, we randomly selected and removed them by sequence, not by message. Although the resulting reduced dataset is much smaller than the original dataset, it is still representative of the original dataset in terms of the distribution of normal and abnormal log messages. Specifically, the original HDFS dataset consists of 11 175629 log messages, with 97.43% normal and 2.57% abnormal log messages, and the reduced HDFS dataset mirrors this distribution, with 97.60% normal and 2.40% abnormal log messages.

**Table 1:** Datasets in LogHub benchmark (He et al. 2020)

Datasets	Anomaly Label	Source Code
Android	✗	✗
Apache	✗	✗
BGL	✓	✗
		
HPC	✗	✗
		
HealthApp	✗	✗
Linux	✗	✗
Mac	✗	✗
OpenSSH	✗	✗
		
Proxifier	✗	✗
Spark	✗	✗
Spirit	✓	✗
Thunderbird	✓	✗
Windows	✗	✗
Zookeeper	✗	✗

**Table 2:** Size information of the log datasets used in our experiments. Number of oracle templates (*O*); Number of all logs ($L_{all}$); Number of normal logs ($L_{n}$); Number of abnormal logs ($L_{a}$); Number of all messages ($M_{all}$); Number of messages in normal logs ($M_{n}$); Number of messages in abnormal logs ($M_{a}$)

Dataset	O	$L_{all}$	$L_{n}$	$L_{a}$	$M_{all}$	$M_{n}$	$M_{a}$
HDFS (reduced)	26	15295	15026	269	299971	292776	7195
Hadoop	175	54	11	43	109968	14392	95576
OpenStack	21	2068	2064	4	79925	79817	108

Table 2 reports on the size of our datasets, in terms of the number of oracle templates (*O*), the number of all logs ($L_{all}$), the number of normal logs ($L_{n}$), the number of abnormal logs ($L_{a}$), the number of all messages ($M_{all}$), the number of messages in normal logs ($M_{n}$), and the number of messages in abnormal logs ($M_{a}$). Note that the number of log messages is the same as the number of log entries (see Section 2.1 for details).

### Log Parsing Techniques

We aimed to use as many log parsing techniques as possible, among those available in the literature. Since (Khan et al. 2022) recently provided a comprehensive evaluation of 14 log parsing techniques (i.e., AEL (Jiang et al. 2008), Drain (He et al. 2017), IPLoM (Makanju et al. 2009), LenMa (Shima 2016), LFA (Nagappan and Vouk 2010), LKE (Fu et al. 2009), LogCluster (Vaarandi and Pihelgas 2015), LogMine (Hamooni et al. 2016), Logram (Dai et al. 2020), LogSig (Tang et al. 2011), MoLFI (Messaoudi et al. 2018), SHISO (Mizutani 2013), SLCT (Vaarandi 2003), and Spell (Du and Li 2016)), we decided to reuse their replication package, including all the aforementioned techniques.

However, we had to exclude LKE since our preliminary evaluation results showed that it could not complete its run for *all* of our log datasets within the 2-day timeout. Notice that we have already reduced our log datasets (in particular, HDFS), as discussed in Section 4.1, based on the slowest anomaly detection technique (i.e., LogAnomaly). Although we could additionally reduce the datasets based on the slowest log parsing technique (i.e., LKE), we found that it would result in small logs that are not representative of the size and complexity of real-world logs.

As a result, we considered 13 log parsing techniques in our experiments. For all the log parsing techniques, we used their default parameters.

### Anomaly Detection Techniques

Similar to the case of log parsing techniques, we considered the work of Le and Zhang (2022), a recent empirical study that evaluated five DL-based anomaly detection techniques (i.e., DeepLog (Du et al. 2017), LogAnomaly (Meng et al. 2019), LogRobust (Zhang et al. 2019), PLELog (Yang et al. 2021), and CNN (Lu et al. 2018)), and decided to use their replication package, including all the aforementioned techniques. For all anomaly detection techniques, we used their default parameters. These techniques are representative of the state of the art of DL-based anomaly detection techniques.

In addition to deep learning models, we included two representative traditional machine learning models, namely Support Vector Machine (SVM) (Hearst et al. 1998) and Random Forest (RF) Breiman (2001)^2^ since they are known for their effectiveness in anomaly detection tasks on the HDFS dataset (Wu et al. 2023; Jia et al. 2023).

We want to note that the seven anomaly detection techniques used in this paper *all require log parsing as a preliminary step*. Although a few recent studies (Le and Zhang 2021; Mvula et al. 2023; Nedelkoski et al. 2020) have proposed anomaly detection techniques that do not require log parsing, we did not consider them in our work. This is mainly because our focus is on assessing the impact of log parsing on anomaly detection techniques. We leave the evaluation of techniques that do not require log parsing for future work.

### Methodology for RQ1

Recall that RQ1 investigates to what extent the accuracy of log parsing affects the accuracy of anomaly detection. To answer RQ1, for each dataset, we first executed the log parsing techniques to generate log parsing results and computed their accuracy in terms of GA, PA, and FTA (see § 2.2). We then executed the anomaly detection techniques on each of the log parsing results and computed their accuracy in terms of precision (PR), recall (RE), and F1 score. By doing so, we obtained a tuple of accuracy values $\langle GA, PA, FTA, PR, RE, F1 \rangle$ for each combination of datasets, log parsing results, and anomaly detection techniques.

For log parsing, we executed each of the log parsing techniques with a 2-day timeout. Since MoLFI is non-deterministic, we executed it three times. In total, we obtained 16 log parsing results (three from the three different executions of MoLFI and 13 from the remaining log parsing techniques) for each dataset. For each log parsing result, we computed $\langle GA, PA, FTA \rangle$ using the oracle templates (and the messages matching them) for the corresponding datasets.

For anomaly detection, we divided the individual log parsing results into two disjoint sets, i.e., a training set and a test set, using a split ratio of 80:20. Considering the data leakage problem mentioned by Le and Zhang (2022), we used the first 80% of the logs (in chronological order) for training and the remaining 20% for testing. We trained the anomaly detection techniques on each of the training sets with a 2-day timeout, and used the corresponding test sets to compute $\langle PR, RE, F1 \rangle$. To account for the randomness of anomaly detection techniques, we repeated the train-and-test process five times and used the average F1 score.

As a result, we obtained 224 tuples $\langle GA, PA, FTA, PR, RE, F1 \rangle$ from the combinations of two datasets, 16 log parsing results, and seven anomaly detection techniques.

### Methodology for RQ2

Recall that RQ2 investigates the relationship between the distinguishability of log parsing results and anomaly detection accuracy. To answer RQ2, we need distinguishable and indistinguishable log parsing results to compare in terms of anomaly detection accuracy. Although the log parsing results generated for RQ1 are available, they are mostly (but not all) distinguishable, leading to unbalanced data for RQ2. To systematically assess the impact of the distinguishability of log parsing results on anomaly detection accuracy using balanced data, we generate pairs of distinguishable and indistinguishable log parsing results.

Specifically, let *d*(*R*) be the distinguishability — expressed as a Boolean value, either *true* (*T*) or *false* (*F*) — of a log parsing result *R*. For each log parsing result *R* (i.e., the result of executing a log parsing technique for a dataset) generated in the context of RQ1 (i.e., 16 log parsing results for each of the two datasets), we first created a pair of log parsing results $$\langle R, R' \rangle $$ by artificially generating $$R'$$ from *R* such that $$d(R') = \lnot d(R)$$ using Algorithms 1 and 2, detailed further below. By definition, if *R* is distinguishable then $$R'$$ will be indistinguishable and vice versa. For the sake of simplicity, we denote the distinguishable result (be it *R* or $$R'$$) as $$R_{dst}$$ and the indistinguishable one (respectively, either $$R'$$ or *R*) as $$R_{ind}$$. We then executed, for all pairs $$\langle R_{dst}, R_{ind} \rangle $$, all the considered anomaly detection techniques twice: the first time using $$R_{dst}$$ as input and the second time using $$R_ ind $$ as input; for each run of each anomaly detection technique we computed its accuracy in terms of precision, recall, and F1 score. By doing so, we obtained the anomaly detection accuracy scores for pairs of distinguishable ($$R_{dst}$$) and indistinguishable ($$R_{ind}$$) versions of log parsing results, and then compared them.

For the generation of $R'$ from *R*, it is important to minimize the difference between *R* and $R'$ (in terms of both training and testing datasets) while achieving $d(R') = \lnot d(R)$. This is to ensure that if there is a difference in anomaly detection scores between *R* and $R'$, it is mostly due to distinguishability and not to other differences between *R* and $R'$ (e.g., the number of templates or the size of log parsing results). Furthermore, the testing datasets for *R* and $R'$ should remain the same. To do this, we need to distinguish the two cases when $d(R) = T$ and when $d(R) = F$, as described below.

#### Generation of Indistinguishable from Distinguishable Log Parsing Results

**Fig. 1:**
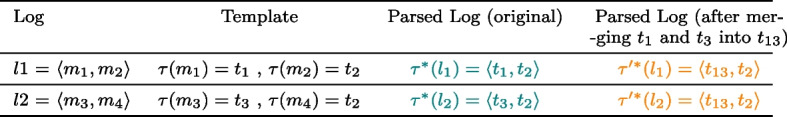
An example of making a distinguishable log parsing result indistinguishable by merging templates

When $d(R) = T$ (i.e., $R = R_{dst}$), it means that templates for different log messages in *R* are different enough to distinguish between normal and abnormal logs in *R*, as explained in Section 2.4. For example, let us consider two logs $l_1 = \langle {m_{1}, m_{2}} \rangle$ and $l_2 = \langle {m_{3}, m_{4}} \rangle$ where the templates of the four messages are identified as $\tau (m_{1}) = t_1$, $\tau (m_{2}) = t_{2}$, $\tau (m_{3}) = t_{3}$, and $\tau (m_{4}) = t_{2}$, respectively, using a log parsing technique $\tau$. Figure 1 shows the logs, messages, and templates. In this case, the log parsing result of $\tau$ for $\{l_1, l_2\}$ is *distinguishable*, as highlighted in blue in the figure, since $\tau ^*(l_{1}) = \langle \tau (m_{1}), \tau (m_{2}) \rangle = \langle t_{1}, t_{2} \rangle$ and $\tau ^*(l_{2}) = \langle {\tau (m_{3}), \tau (m_{4})} \rangle = \langle t_{3}, t_{2} \rangle$ are different (due to $\tau (m_{1}) \ne \tau (m_{3})$, i.e., $t_1 \ne t_{3}$). However, if the templates of $m_{1}$ and $m_3$ were the same, then the log parsing result would be *indistinguishable*. In other words, as highlighted in red in the figure, we can make the distinguishable log parsing result of $\tau$ indistinguishable by merging the templates of $m_{1}$ and $m_{3}$ (e.g., by introducing a dummy log parsing technique $\tau '$ that behaves the same as $\tau$ except for $\tau '(m_{1}) = \tau '(m_{3}) = t_{13}$). Notice that $\tau '$ changes only (a few) templates, not the corresponding log messages, meaning that the original datasets remain the same. Using this idea, to generate $R' = R_{ind}$ from $R = R_{dst}$, we generated the templates of $R_{ind}$ by iteratively merging the templates of $R_{dst}$ until $d(R_{ind}) = F$. Furthermore, to minimize the difference between $R_{dst}$ and $R_{ind}$ in terms of the number of templates (i.e., to minimize the number of templates being merged), we start with merging the templates with the highest number of matching messages in the log. This is based on the intuition that the more messages affected by merging templates, the more likely normal and abnormal logs are to become indistinguishable. Recall that we only change the templates, not their log messages.

Although merging templates to generate indistinguishable log parsing results might look artificial, it is indeed realistic to some extent. In practice, a log parsing result would be indistinguishable only when a log parsing technique fails to identify proper templates that can sufficiently “distinguish” normal and abnormal log sequences. Therefore, merging templates in the distinguishable log parsing results mimics the behavior of such imperfect log parsing techniques, leading to indistinguishable log parsing results.

One might also object that artificially merging templates corresponding to different messages could introduce incorrect templates in $R_{ind}$, leading to an unfair comparison between $R_{dst}$ and $R_{ind}$. However, it is common for the log parsing techniques to identify many templates that are already incorrect Khan et al. (2022). Furthermore, the focus of RQ2 is not the correctness of templates but rather the distinguishability of log parsing results. Our goal is to generate a pair of $R_{dst}$ and $R_{ind}$ that are as similar as possible except for the distinguishability property. Indeed, the testing datasets for $R_{dst}$ and $R_{ind}$ are the same in terms of log messages and their order. The only difference lies in how individual log messages are mapped to the templates, affecting the distinguishability of log parsing results. Consequently, the only difference between $R_{dst}$ and $R_{ind}$ is in their distinguishability, ensuring that no bias is introduced when evaluating the model’s performance.

Algorithm 1 summarizes the above-mentioned idea into the pseudocode for generating $R_{ind}$ from $R_{dst}$. After initializing $R_{ind}$ (line 1) as a copy of $R_{dst}$, the algorithm extracts the set of templates *T* of $R_{dst}$ (line 2) and sorts the templates in *T* in ascending order by the number of matching messages (line 3). The algorithm then iteratively merges the last *n* templates (starting from $n=2$ as initialized at line 4) in the sorted templates list $T_s$ (i.e., merging the top-*n* templates that have the highest number of matching templates) until $R_{ind}$ becomes indistinguishable (lines 5–8). Notice that the while loop does not continue endlessly since $R_{ind}$ must be indistinguishable when *n* becomes $|T_s|$ (i.e., all templates are merged into one) by definition. The algorithm ends by returning $R_{ind}$.

**Algorithm 1:**
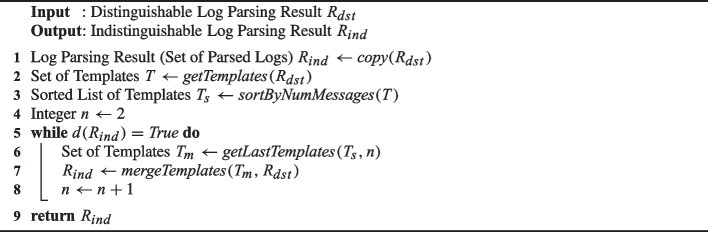
Generating an indistinguishable log parsing result from a distinguishable one

#### Generation of Distinguishable from Indistinguishable Log Parsing Results

When $d(R) = F$ (i.e., $R = R_{ind}$), although one could do the dual of merging templates (i.e., dividing templates), it would require to determine which templates to divide and how many templates to generate from a given template. Instead, we adopted another heuristic: we removed the normal (or abnormal) logs that are indistinguishable from abnormal (or normal) logs. This is based on our observation that, when $d(R) = F$, only a small number of normal and abnormal logs are indistinguishable. To minimize the impact of removing logs, we removed normal logs when the total number of normal logs is larger than that of abnormal logs (as it is the case for the HDFS dataset); otherwise, we removed abnormal logs (in the case of the Hadoop dataset). Specifically, only MoLFI, SLCT, LogCluster, and LFA generated indistinguishable log parsing results for HDFS in the first place, and we only removed 5, 5, 9, and 2 logs, respectively, out of 15026 normal logs.

Algorithm 2 shows how to generate $R_{dst}$ from $R_{ind}$ based on the above idea. It first extracts the set of indistinguishable logs $L_{ind}$ from $R_{ind}$ (line 1). It then removes either normal or abnormal logs in $L_{ind}$ from $R_{ind}$ to generate $R_{dst}$ depending on the total number of normal and abnormal logs (lines 2–5). Since $R_{dst}$ is the result of removing indistinguishable (normal or abnormal) logs from $R_{ind}$, $R_{dst}$ is distinguishable. The algorithm ends by returning $R_{dst}$.

**Algorithm 2:**
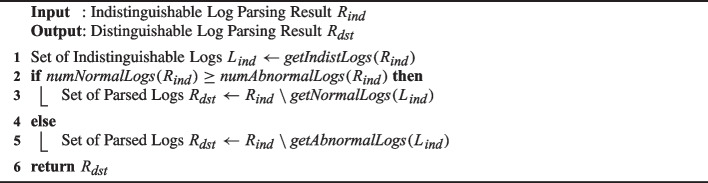
Generating a distinguishable log parsing result from an indistinguishable one

#### Treatment for Anomaly Detection Techniques using Semantic Information of Templates

Some of the anomaly detection techniques (i.e., LogRobust (Zhang et al. 2019), PLELog (Yang et al. 2021), LogAnomaly (Meng et al. 2019)) use the semantic information of templates, instead of simply using template IDs, by converting them into semantic vectors (Jurafsky and Martin 2019). For these techniques, two templates are considered “identical” if their semantic vectors are similar enough. Therefore, the notion of “identical” templates for determining the distinguishability of log parsing results must be revised in terms of the semantic vectors used by these anomaly detection techniques; otherwise, simply determining the distinguishability based on their template IDs would be meaningless for these techniques. To do this, for each log parsing result *R*, we applied a clustering algorithm to the semantic vectors of all templates and considered the templates in the same cluster to be identical. Specifically, we used DBSCAN (Backlund et al. 2011) for clustering since it does not require the number of clusters as an input parameter. For instance, in the above example $\tau$ with $m_1$ and $m_3$, if the semantic vectors of $\tau (m_1)$ and $\tau (m_3)$ belong to the same cluster, then the templates of $m_1$ and $m_3$ are considered the same. Note that the semantic vectors are carefully designed to capture subtle semantic nuances and are able to identify semantically similar log templates while distinguishing different ones (Zhang et al. 2019). Therefore, clustering these semantic vectors can effectively identify “identical” templates for the semantic-based anomaly detection techniques. We then followed the same heuristics described above to generate $R'$ from *R* based on the clustered templates.

#### Additional Analysis: Degree of Distinguishability

So far, we have described how to compare distinguishable and indistinguishable log parsing results to answer RQ2, treating distinguishability as a binary property (i.e., either distinguishable or indistinguishable) following the original definition (Shin et al. 2021). Although we have effectively minimized the difference between distinguishable and indistinguishable log parsing results to make a fair comparison, we have applied an *artificial* process for generating indistinguishable log parsing results from distinguishable ones (or vice versa). To address this limitation, we present an additional analysis on the degree of distinguishability of the log parsing results generated for RQ1.

However, defining a metric to measure the degree of distinguishability is not straightforward, mainly because the original definition of distinguishability is too strict; for example, the log parsing result of two log sequences representing the same behavior can be considered distinguishable simply when they are different in length. Therefore, we present a metric to measure the degree of distinguishability based on the number of common templates between normal and abnormal log sequences. This is based on the observation that a higher number of shared templates between normal and abnormal log sequences indicates weaker distinguishability.

Specifically, recall that we can consider a log parsing result $$\tau ^{**}(L)$$ of a set of log sequences *L* for a log parsing technique $$\tau $$. Let $$c(\tau ^{**}(L))$$ be the number of unique templates in $$\tau ^{**}(L)$$. We define the distinguishability score $$\textit{distScore}(\tau , L)$$ of *L* for $$\tau $$ as the ratio of the number of common templates generated by $$\tau $$ between normal and abnormal log sequences to the number of unique templates in all log sequences in *L*, i.e., $$\textit{distScore}(\tau , L) = 1 - \frac{c(\tau ^{**}(L_n) \cap \tau ^{**}(L_a))}{c(\tau ^{**}(L))}$$, where $$L_n$$ and $$L_a$$ are the sets of normal and abnormal log sequences in *L*, respectively. Since $$c(\tau ^{**}(L)) = c(\tau ^{**}(L_n)) \cup c(\tau ^{**}(L_a))$$, the distinguishability score is effectively the Jaccard distance between $$L_n$$ and $$L_a$$ in terms of their templates. For example, the number of unique templates identified by Drain for the HDFS dataset is 31. Among them, 13 templates appear in both normal and abnormal log sequences. Therefore, the distinguishability score of Drain for the HDFS dataset is $$1 - \frac{13}{31} = 0.57$$.

We want to note that, ideally speaking, this additional analysis should allow us to measure the impact of distinguishability on anomaly detection accuracy in a more fine-grained manner without generating artificial log parsing results. However, our metric is a heuristic and may not fully capture the various aspects of distinguishability. Therefore, we will use this new analysis as a complementary study to the main analysis (treating distinguishability as a binary property), to provide a more comprehensive understanding of the impact of distinguishability on anomaly detection accuracy.

## Results

### RQ1: Relationship between Log Parsing Accuracy and Anomaly Detection Accuracy

All 13 log parsing techniques and 7 anomaly detection techniques completed their executions on the HDFS and Hadoop datasets. However, none of the anomaly detection techniques detected abnormal logs in the OpenStack dataset (i.e., the F1 score is zero). This could be due to the very small number of abnormal logs in the dataset (only 4 out of 2068, as reported in Table 2). Therefore, we disregard the results for OpenStack.

**Fig. 2:**
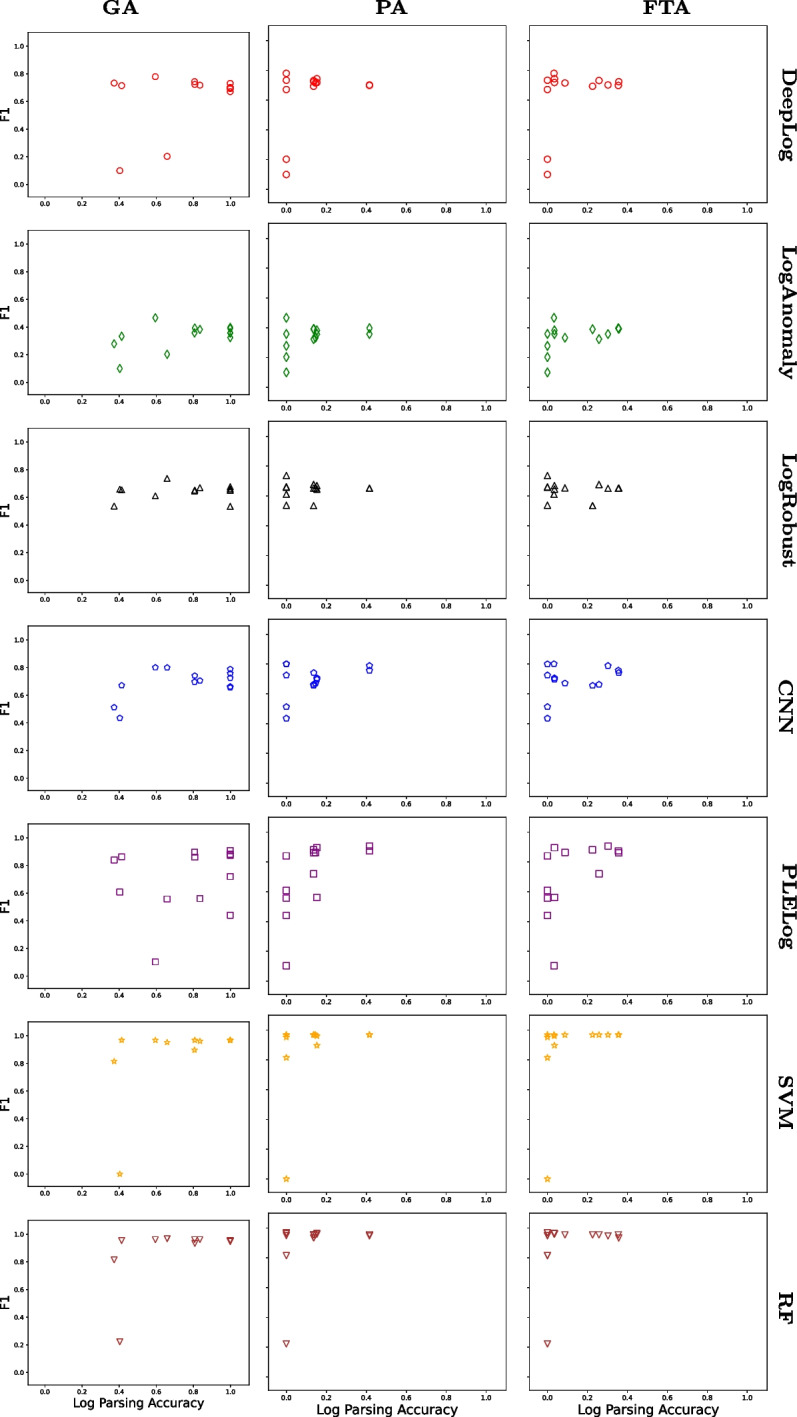
Relationship between TI accuracy and AD accuracy (HDFS)

**Fig. 3:**
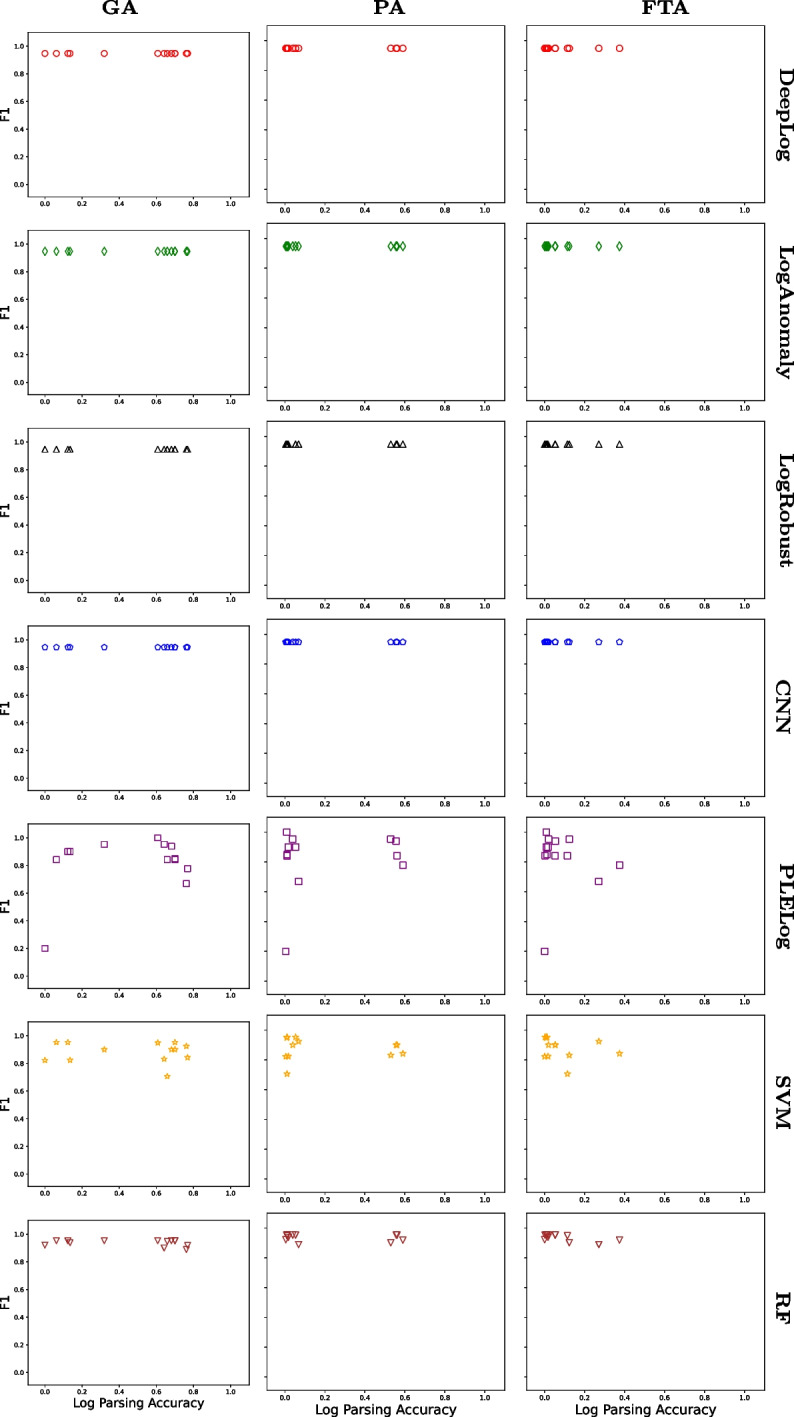
Relationship between TI accuracy and AD accuracy (Hadoop)

For all tuples $\langle {GA, PA, FTA, PR, RE, F1} \rangle$ we collected for HDFS and Hadoop, Figs. 2 and 3 show the relationship between $\langle {GA, PA, FTA} \rangle$ (x-axis) and *F*1 (y-axis) for HDFS and Hadoop, respectively, in the form of a scatter plot. To additionally distinguish the main results for different anomaly detection techniques, we used different shapes and colors:  = DeepLog,  = LogAnomaly,  = LogRobust,  = CNN,  = PLELog,  = SVM, and  = RF. For example, the top left subfigure in Fig. 2 shows 13 data points where 13 log parsing techniques are used in combination with DeepLog. All the raw data are available in the replication package on Figshare Khan et al. (2024).

Table 3 additionally shows the values of the Spearman’s rank correlation coefficient $\sigma \langle {X, Y} \rangle$ between $X= \langle {GA, PA, FTA} \rangle$ and $Y= F1$ for each pair of anomaly detection technique and dataset. The value of $\sigma \langle {X, Y} \rangle$, ranging between $-1$ and $+1$, is an indication of the strength of the monotonic (not necessarily linear) relationship between *X* and *Y*; when $\sigma \langle {X, Y} \rangle \ge +0.7$ (or $\sigma \langle {X, Y} \rangle \le -0.7$), there is a *strong* positive (or negative) correlation between *X* and *Y* Ali Abd Al-Hameed (2022). Note that, on the Hadoop dataset, $\sigma \langle {X, Y} \rangle$ could not be computed for DeepLog, LogAnomaly, LogRobust, and CNN since the F1 score does not vary at all with $\langle {GA, PA, FTA} \rangle$, indicating no relationship.

**Table 3:** Spearman correlation coefficients between log parsing accuracy (GA, PA, and FTA) and anomaly detection accuracy (F1 score)

AD technique	HDFS (reduced)			Hadoop		
	GA	PA	FTA	GA	PA	FTA
DeepLog	$-$0.166	0.259	0.198	–	–	–
LogAnomaly	0.431	0.455	0.527	–	–	–
LogRobust	0.216	$-$0.134	$-$0.162	–	–	–
CNN	0.276	0.262	0.195	–	–	–
PLELog	0.171	0.656	0.628	$-$0.180	$-$0.003	$-$0.069
SVM	0.633	0.371	0.650	$-$0.011	0.053	$-$0.346
RF	0.118	0.205	$-$0.063	$-$0.303	–0.136	$-$0.569

Overall, Figs. 2, 3, and Table 3 clearly show that there is no strong correlation between $\langle {GA, PA, FTA} \rangle$ and *F*1 in all the cases where $\langle {GA, PA, FTA, PR, RE, F1} \rangle$ tuples were successfully collected. For example, in Fig. 2, LogAnomaly (  ) achieved an F1 score ranging between 0.2 and 0.5 regardless of the GA score. This means that increasing log parsing accuracy does not necessarily increase (or decrease) anomaly detection accuracy. This is counter-intuitive since anomaly detection uses log parsing results, and having “better” log parsing results is expected to increase anomaly detection accuracy. However, this happens because even inaccurate log parsing results can lead to accurate anomaly detection results, for reasons explained below.

To better understand the reason for the above results, let us consider the following two extreme cases separately:

1. The log parsing accuracy values for input logs are the same, but the resulting anomaly detection accuracy values are different (i.e., the data points located on the same vertical lines in Figs. 2 and 3).
2. The log parsing accuracy values for input logs are different, but the resulting anomaly detection accuracy values are the same (i.e., the data points located on the same horizontal lines in Figs. 2 and 3).

To identify the root cause of C1, we manually investigated several pairs of data points in Figs. 2 and 3, such as two different HDFS log parsing results having almost the same log parsing accuracy value (GA scores of 0.37 and 0.40) but resulting in significantly different anomaly detection accuracy values (F1 scores of 0.73 and 0.10) for the same anomaly detection technique (DeepLog). It turned out that, although the log parsing accuracy values are similar, the sets of correctly parsed log messages are different. This happened because the log parsing accuracy metrics (GA, PA, and FTA) summarize the log parsing results based on an implicit assumption that all log messages (and templates) are equally important. However, this assumption does not hold when it comes to anomaly detection, which must discriminate different log message templates to learn abnormal sequences of templates. Therefore, this mismatch of assumptions between log parsing and anomaly detection leads to case C1.

As for case C2, similar to the above case, we manually investigated several pairs of data points in Figs. 2 and 3, such as two different Hadoop log parsing results having significantly different log parsing accuracy values (GA scores of 0.12 and 0.77) but resulting in the same anomaly detection value (F1 score of 0.98) for the same anomaly detection technique (DeepLog). We found that anomaly detection techniques can distinguish between normal and abnormal patterns even when input log message templates are incorrect. To best explain this using a simplified example, let us consider a normal log $l_n = \langle {m_1^n, m_2^n}, \dots \rangle$ and an abnormal log $l_a = \langle {m_1^a, m_2^a}, \dots \rangle$, where $m_i^x$ indicates the *i*-th log message in $l_x$ for $x\in \{n, a\}$. Using oracle templates, we can group the log messages having the same template and represent $l_n$ and $l_a$ as groups; specifically, let $g_{orc}(l_x)$ be a sequence of message group indices (i.e., the *i*-th element of $g_{orc}(l_x)$ is the message group index of $m_i^x$). In this context, let us take two logs from the Hadoop dataset as a concrete example where $g_{orc}(l_n) = \langle 1, 2, 3, 4, \dots \rangle$ and $g_{orc}(l_a) = \langle 5, 5, 5, 6, \dots \rangle$. When templates generated by LogMine are used to group messages instead of oracle templates, the sequences of message group indices change to $g_{LM}(l_n) = \langle 1, 2, 3, 3, \dots \rangle$ and $g_{LM}(l_a) = \langle 7, 8, 9, 10, \dots \rangle$. These are clearly different from $g_{orc}(l_n)$ and $g_{orc}(l_a)$, respectively; in particular, $m_3^n$ and $m_4^n$ are incorrectly grouped together in $g_{LM}(l_n)$ while $m_1^a$, $m_2^a$, and $m_3^a$ are incorrectly separated in $g_{LM}(l_a)$. The incorrect groupings of LogMine clearly reduce the GA score (as well as PA and TA scores since incorrect groupings imply incorrect templates). However, even the incorrect $g_{LM}(l_n)$ and $g_{LM}(l_a)$ are still different enough from each other for anomaly detection techniques to distinguish between normal and abnormal patterns. This example not only shows why case C2 happened, but also demonstrates the importance of *distinguishability* in log parsing results for anomaly detection; we will further investigate this aspect in RQ2.

Before we conclude RQ1, one might be curious to know why DeepLog, LogAnomaly, LogRobust, and CNN result in the same anomaly detection accuracy value on the Hadoop dataset (as shown in Fig. 3 [GA-Hadoop] and Table 3). This happens because (1) the test set of Hadoop contains only 11 logs (1 normal and 10 abnormal logs, although the number of log messages is in the same order of magnitude as HDFS; see Table 2 for more details) and (2) the four anomaly detection techniques classified all the 11 logs in the test set as abnormal. We speculate that PLELog shows different results from the other anomaly detection techniques because PLELog uses a very different deep learning model (i.e., an attention-based GRU (Cho et al. 2014)). Notice that, in all cases, the results still corroborate that log parsing accuracy and anomaly detection accuracy do not have any strong relationship.

We want to note that the log parsing accuracy results shown in Figs. 2 and 3 are inconsistent with the ones reported in previous studies Zhu et al. (2019); Dai et al. (2020) since the latter only considered 2K log messages, randomly sampled from the original logs, to assess log parsing accuracy.

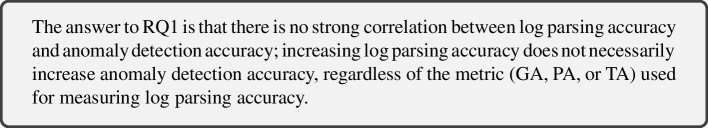



**Table 4:** Impact of the distinguishability log parsing results on anomaly detection accuracy for the HDFS (reduced) dataset (DL-based anomaly detection techniques)

Log Parser	DeepLog (F1)			LogAnomaly (F1)			LogRobust (F1)			CNN (F1)			PLELog (F1)		
	$R_{dst}$	$R_{ind}$	$\Delta$	$R_{dst}$	$R_{ind}$	$\Delta$	$R_{dst}$	$R_{ind}$	$\Delta$	$R_{dst}$	$R_{ind}$	$\Delta$	$R_{dst}$	$R_{ind}$	$\Delta$
AEL	0.747	0.561	0.186	0.509	0.320	0.189	0.663	0.456	0.207	0.772	0.662	0.110	0.760	0.033	0.727
Drain	0.714	0.523	0.191	0.499	0.400	0.099	0.703	0.454	0.250	0.757	0.682	0.075	0.796	0.286	0.510
IPLoM	0.760	0.590	0.170	0.481	0.268	0.213	0.556	0.380	0.176	0.810	0.588	0.222	0.849	0.041	0.808
LFA	0.803	0.693	0.110	0.606	0.378	0.228	0.355	0.299	0.056	0.755	0.548	0.207	0.100	0.000	0.100
LenMa	0.808	0.625	0.184	0.484	0.285	0.199	0.659	0.436	0.223	0.814	0.607	0.207	0.681	0.271	0.411
LogCluster	0.263	0.097	0.166	0.380	0.243	0.138	0.542	0.300	0.241	0.498	0.306	0.192	0.426	0.317	0.108
LogMine	0.732	0.552	0.180	0.453	0.363	0.090	0.554	0.329	0.225	0.792	0.612	0.179	0.817	0.439	0.378
Logram	0.202	0.025	0.177	0.290	0.143	0.148	0.696	0.460	0.236	0.699	0.523	0.176	0.787	0.034	0.753
MoLFI	0.794	0.630	0.164	0.427	0.282	0.144	0.565	0.319	0.246	0.781	0.621	0.160	0.172	0.109	0.063
SHISO	0.778	0.629	0.149	0.544	0.238	0.306	0.679	0.446	0.233	0.796	0.589	0.207	0.839	0.341	0.498
SLCT	0.743	0.570	0.173	0.268	0.160	0.108	0.394	0.244	0.150	0.743	0.607	0.136	0.725	0.534	0.191
Spell	0.765	0.598	0.167	0.289	0.176	0.113	0.401	0.241	0.160	0.805	0.616	0.189	0.665	0.304	0.361
Average	0.676	0.508	0.168	0.436	0.271	0.164	0.564	0.364	0.200	0.752	0.580	0.172	0.635	0.226	0.409

**Table 5:** Impact of the distinguishability of log parsing results on anomaly detection accuracy for the HDFS (reduced) dataset (ML-based anomaly detection techniques)

Log Parser	SVM (F1)			RF (F1)		
	$R_{dst}$	$R_{ind}$	$\Delta$	$R_{dst}$	$R_{ind}$	$\Delta$
AEL	0.968	0.928	0.040	0.947	0.679	0.269
Drain	0.968	0.928	0.040	0.954	0.679	0.276
IPLoM	0.968	0.928	0.040	0.961	0.679	0.282
LFA	0.968	0.928	0.040	0.947	0.679	0.269
LenMa	0.968	0.928	0.040	0.947	0.679	0.269
LogCluster	0.000	0.000	0.000	0.222	0.000	0.222
LogMine	0.947	0.928	0.019	0.947	0.782	0.165
Logram	0.868	0.708	0.160	0.968	0.602	0.366
MoLFI	0.968	0.928	0.040	0.959	0.679	0.280
SHISO	0.961	0.928	0.033	0.940	0.679	0.262
SLCT	0.968	0.928	0.040	0.940	0.679	0.262
Spell	0.968	0.928	0.040	0.947	0.679	0.269
Average	0.876	0.832	0.044	0.890	0.624	0.266

**Table 6:** Impact of the distinguishability of log parsing results on anomaly detection accuracy for the Hadoop dataset (DL-based anomaly detection techniques)

Log Parser	DeepLog (F1)			LogAnomaly (F1)			LogRobust (F1)			CNN (F1)			PLELog (F1)		
	$R_{dst}$	$R_{ind}$	$\Delta$	$R_{dst}$	$R_{ind}$	$\Delta$	$R_{dst}$	$R_{ind}$	$\Delta$	$R_{dst}$	$R_{ind}$	$\Delta$	$R_{dst}$	$R_{ind}$	$\Delta$
AEL	0.947	0.847	0.100	0.947	0.847	0.100	0.947	0.847	0.100	0.947	0.847	0.100	0.785	0.507	0.278
Drain	0.947	0.847	0.100	0.947	0.847	0.100	0.947	0.847	0.100	0.947	0.847	0.100	0.900	0.000	0.900
IPLoM	0.947	0.847	0.100	0.947	0.847	0.100	0.947	0.847	0.100	0.947	0.847	0.100	0.848	0.000	0.848
LFA	0.947	0.847	0.100	0.947	0.847	0.100	0.947	0.847	0.100	0.947	0.847	0.100	0.888	0.799	0.090
LenMa	0.947	0.847	0.100	0.947	0.847	0.100	0.947	0.847	0.100	0.947	0.847	0.100	0.900	0.505	0.395
LogCluster	0.947	0.847	0.100	0.947	0.847	0.100	0.947	0.847	0.100	0.947	0.847	0.100	0.952	0.180	0.772
LogMine	0.947	0.847	0.100	0.947	0.847	0.100	0.947	0.847	0.100	0.947	0.847	0.100	0.848	0.530	0.318
Logram	0.947	0.847	0.100	0.947	0.847	0.100	0.947	0.847	0.100	0.947	0.847	0.100	0.842	0.799	0.043
MoLFI	0.947	0.847	0.100	0.947	0.847	0.100	0.947	0.847	0.100	0.947	0.847	0.100	0.900	0.739	0.161
SHISO	0.947	0.847	0.100	0.947	0.847	0.100	0.947	0.847	0.100	0.947	0.847	0.100	0.900	0.000	0.900
SLCT	0.947	0.847	0.100	0.947	0.847	0.100	0.947	0.847	0.100	0.947	0.847	0.100	0.842	0.000	0.842
Spell	0.947	0.847	0.100	0.947	0.847	0.100	0.947	0.847	0.100	0.947	0.847	0.100	0.703	0.188	0.515
Average	0.947	0.847	0.100	0.947	0.847	0.100	0.947	0.847	0.100	0.947	0.847	0.100	0.859	0.354	0.505

**Table 7:** Impact of the distinguishability of log parsing results on anomaly detection accuracy for the Hadoop dataset (ML-based anomaly detection techniques)

Log Parser	SVM (F1)			RF (F1)		
	$R_{dst}$	$R_{ind}$	$\Delta$	$R_{dst}$	$R_{ind}$	$\Delta$
AEL	0.960	0.912	0.048	0.952	0.718	0.234
Drain	0.952	0.912	0.040	0.910	0.862	0.047
IPLoM	0.949	0.912	0.037	0.891	0.862	0.029
LFA	0.940	0.912	0.027	0.901	0.862	0.038
LenMa	0.952	0.912	0.040	0.952	0.718	0.234
LogCluster	0.952	0.912	0.040	0.952	0.718	0.234
LogMine	0.960	0.912	0.048	0.952	0.718	0.234
Logram	0.952	0.852	0.100	0.798	0.643	0.155
MoLFI	0.949	0.912	0.037	0.952	0.718	0.234
SHISO	0.924	0.912	0.011	0.936	0.862	0.074
SLCT	0.937	0.912	0.024	0.936	0.862	0.074
Spell	0.960	0.912	0.048	0.952	0.718	0.234
Average	0.949	0.907	0.042	0.924	0.772	0.152

### RQ2: Log Parsing Distinguishability and Anomaly Detection Accuracy

#### Distinguishability as a Binary Property

Tables 4 and 5 show the anomaly detection accuracy values (F1 scores) when different log parsing techniques (rows) and anomaly detection techniques (columns) are used together on the HDFS (reduced) dataset; under each of the anomaly detection technique columns, sub-columns $R_{dst}$ and $R_{ind}$ indicate the F1 scores for distinguishable and indistinguishable log parsing results, respectively, and $\Delta$ indicates the difference between $R_{dst}$ and $R_{ind}$. For example, if we choose AEL for log parsing and DeepLog for anomaly detection, the F1 score decreases from 0.747 to 0.561 when $R_{ind}$ is used instead of $R_{dst}$. The same structure applies to Tables 6 and 7, which show the results on the Hadoop dataset. In Table 6, except for PLELog, SVM, and RF, the values for all anomaly detection techniques are identical due to the reasons explained in the last paragraph of Section 5.1. We do not provide results for the OpenStack dataset due to the reasons mentioned in Section 5.1.

In all cases, $\Delta$ is non-negative, ranging from 0 (LogCluster-SVM on the HDFS dataset) to 0.9 (Drain/SHISO-PLELog on the Hadoop dataset). This means that the anomaly detection accuracy decreases up to 90 percentage points (pp) when $R_{ind}$ is used instead of $R_{dst}$. To see if the differences between $R_{dst}$ and $R_{ind}$ are significant, we applied the non-parametric Wilcoxon signed rank test (Wilcoxon 1992) for paired samples to the F1 scores of $R_{dst}$ and $R_{ind}$, for each of the seven anomaly detection techniques and the two datasets. The results show that, for all the anomaly detection techniques and datasets, the differences between $R_{dst}$ and $R_{ind}$ are significant ($p\text {-value} < 0.005$) in terms of anomaly detection accuracy.

Considering the definition of distinguishability for log parsing results, it is intuitive that indistinguishable log parsing results should lead to lower anomaly detection accuracy. However, it is surprising that this decrease in accuracy is, in some cases, rather limited, e.g., only 0.011 for SHISO on the Hadoop dataset when SVM is used for log parsing. This happens because an indistinguishable log parsing result may only have a few logs that are indistinguishable in terms of normal and abnormal behavior. Recall that we did not explicitly control the number of indistinguishable logs since we aimed to minimize the difference between distinguishable and indistinguishable versions of each log parsing result as described in Section 4.5. Nevertheless, the results shown in Tables 4 and 6 are sufficient to confirm the strong impact of distinguishability in log parsing results on anomaly detection accuracy.

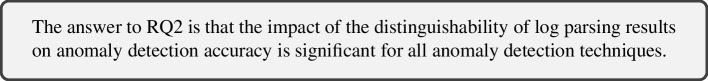



#### Degree of Distinguishability

As explained in Section 4.5.4, let us consider the degree of distinguishability of the log parsing results generated for RQ1 (without considering the artificially generated pairs of $R_{dst}$ and $R_{ind}$.) We focus on the HDFS dataset for this analysis since we know from the RQ1 results that (1) none of the anomaly detection techniques detected abnormal logs in the OpenStack dataset, and (2) most of the anomaly detection techniques have achieved the same accuracy on the Hadoop dataset. Nevertheless, to avoid drawing conclusions based on a single dataset, we also include another dataset, BGL, in this analysis. Although it was excluded from the previous analyses due to the unavailability of source code (which is essential to measure log parsing accuracy), it can be used to investigate the relationship between the degree of distinguishability and anomaly detection accuracy. To use the BGL dataset, we first reduced it following the same methodology we used for the other datasets (see Section 4.1). Since the dataset has only one extremely long normal log, we created log sequences using a sliding window with a window size of 10, following existing studies (Yang et al. 2021; Le and Zhang 2022). We then labelled each log sequence as normal or abnormal as follows: If a log sequence contains at least one abnormal log message, it is considered abnormal; otherwise, it is considered normal. In total, we used 275 306 normal and 16 413 abnormal log sequences from the BGL dataset.

**Fig. 4:**
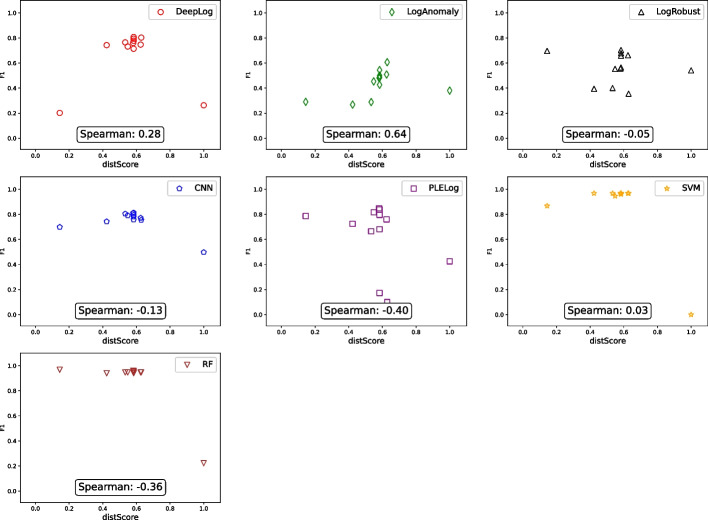
Relationship between *distScore* and AD Accuracy (HDFS)

*HDFS dataset* Figure 4 shows the relationship between the degree of distinguishability (i.e., the *distScore*, shown in the x-axis) and the anomaly detection accuracy (i.e., the F1-score, shown in the y-axis) for the HDFS dataset. Each sub-figure corresponds to a different anomaly detection technique, and each data point represents a log parsing technique. The Spearman correlation coefficient between the *distScore* and the F1-score is also shown in each sub-figure. For DeepLog, LogAnomaly, LogRobust, and CNN, the F1-score mostly increases with the distinguishability score, except for an outlier around ${distScore} = 0.99$. This means that the anomaly detection accuracy mostly improves when the log parsing results are more distinguishable, except for the outlier. This outlier is due to LogCluster, which generates an exceptionally high number of templates, 39 998, while the number of oracle templates is only 26 as noted in Table 2. Although such a large number of templates leads to a high degree of distinguishability between normal and abnormal log sequences due to the high specificity of the templates, it also leads to an excessive number of “features” to consider for the learning-based anomaly detection techniques, making the learning from training data more difficult, resulting in decreased anomaly detection accuracy. For the ML-based anomaly detection techniques, i.e., SVM and RF, the F1-score remains similar regardless of the distinguishability score, except for the same outlier discussed above. We suspect that this is mainly because the traditional ML-based techniques are more sensitive to the number of features they use for learning (i.e., the number of templates, which typically range from 26 to 201) than to the degree of distinguishability. However, LogCluster notably identifies a significantly higher number of templates, totaling 39 998. For PLELog, the F1-score does not show a clear correlation with the distinguishability score. This could be mainly due to the unique architecture of PLELog, which uses Gated Recurrent Units (GRUs) to model the log sequences, as discussed in Section 5.1.

**Fig. 5:**
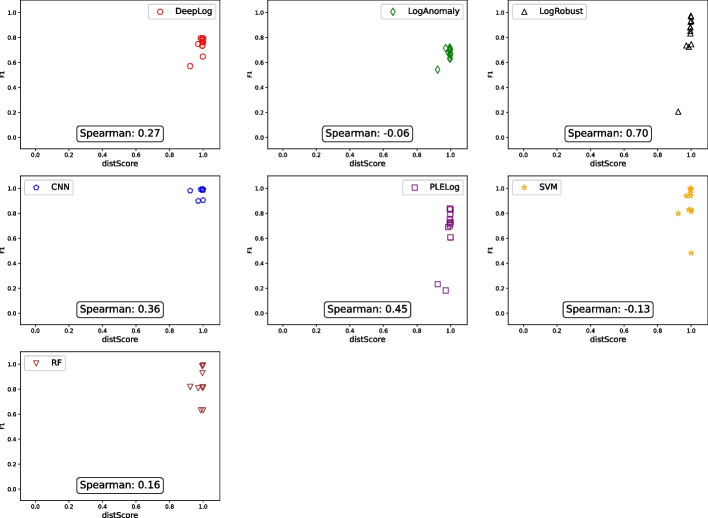
Relationship between *distScore* and AD Accuracy (BGL)

*BGL dataset* Figure 5 shows the results for the BGL dataset. The structure of the figure is the same as that of Fig. 4. Overall, the F1-score mostly increases with the distinguishability score, except for LogAnomaly and SVM. However, their Spearman correlations are very weak (only $-0.06$ and $-0.13$, respectively). In other cases, the Spearman correlations are positive, ranging from 0.16 (RF) to 0.70 (LogRobust). This implies that the findings from the HDFS dataset are generally consistent with those from the BGL dataset.

To sum up, although the degree of distinguishability of log parsing results is not always positively related to anomaly detection accuracy, most of the deep learning-based techniques show moderate and positive correlations between the distinguishability degree and the anomaly detection accuracy. Considering the heuristic nature of the proposed distinguishability score, defining a more sophisticated and precise metric that can better capture the relationship between the distinguishability of log parsing results and the anomaly detection accuracy is an interesting direction for future work.

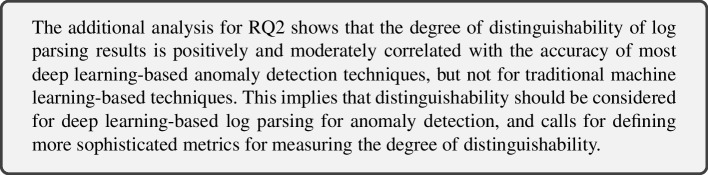



### Threats to Validity

The used oracle templates determine log parsing accuracy values. For example, as noted by Khan et al. (2022), manually extracting oracle templates by investigating log messages without accessing the corresponding source code could result in biased, incorrect oracle templates. This could be a significant threat to the validity of our results. To mitigate this, we perused the source code (of the exact version that generated the logs) for each software system and used the templates directly extracted from the source code. Although this made us exclude a few log datasets whose source code was unavailable, it was beneficial to ensure the validity of our results.

Individual log parsing and anomaly detection techniques have distinct hyper-parameters, which might significantly affect the log parsing and anomaly detection results. To mitigate this, we used the same hyper-parameter values proposed by the authors, when available; otherwise, we ran preliminary experiments and used the values that resulted in the same results reported in the corresponding papers.

Using a specific set of log datasets is a potential threat to external validity. Though the datasets we considered include the logs of various systems, we had to select HDFS, Hadoop, and OpenStack due to the reasons discussed in Section 4.1. Therefore, even though the datasets have been widely used in existing literature Le and Zhang (2022); Chen et al. (2021) on log-based anomaly detection, they may not capture diverse characteristics of log data. Further experiments with different datasets are required to improve the generalizability of our results.

In RQ2, we artificially generated pairs of distinguishable and indistinguishable log parsing results to systematically assess the impact of the distinguishability of log parsing results on anomaly detection accuracy using balanced data. To mitigate any bias introduced during the process, we carefully designed Algorithms 1 and 2 to minimize the difference between each pair of log parsing results, except for their distinguishability property. Note that, although the pair generation process (by merging templates) might look unrealistic, it reflects what frequently happens in real-world scenarios; for example, it is not uncommon for log parsing techniques to misidentify templates so that messages with different oracle templates are mapped to the same (misidentified) template.

## Findings and Implications

One of the most surprising results from our evaluation is that, using all existing log parsing accuracy metrics in the literature, we did not find any significant correlation with anomaly detection accuracy. In other words, more accurate log parsing results are not necessarily better for anomaly detection accuracy. This implies that log parsing accuracy is not a good indicator of the quality of log parsing results for anomaly detection purposes. As explained with an example in Section 5.1, this happens because inaccurate log parsing results can still be useful for anomaly detection as long as normal and abnormal logs are distinguishable. At the extreme, a log parsing result $R_{50}$ with 50% accuracy could be better for anomaly detection than a log parsing result $R_{100}$ with 100% accuracy if $R_{50}$ distinguishes normal and abnormal logs while $R_{100}$ does not. This could happen when, for example, the log quality is poor (e.g., because of inconsistencies between the developers’ intentions and concerns on logging and the actual logging statements in the source code (Rong et al. 2020)) to the point that even using oracle templates cannot fully distinguish all normal log sequences from abnormal ones.

This surprising finding leads to an important practical implication: When used for anomaly detection purposes, we can no longer choose a log parsing technique based on accuracy. Instead, as shown in Section 5.2, the distinguishability of log parsing results should be the main selection criterion. For example, since normal and abnormal logs are often used for training anomaly detection models, candidate log parsing results should be compared in terms of their capability to distinguish normal and abnormal logs. If there are multiple techniques that can equally distinguish between normal and abnormal logs, then the one with the lowest number of identified templates would be preferred since reducing the number of templates would increase the performance of anomaly detection by reducing dimensionality (i.e., the number of features considered in machine learning models) Shin et al. (2021).

Note that the notion of distinguishability for log parsing results is irrelevant if these results are not used for anomaly detection. However, if anomaly detection needs log parsing (which is frequently the case in practice), then considering distinguishability can help engineers select the most suitable log parsing technique for anomaly detection.

One may rightfully think that it is intuitive that the distinguishability of log parsing results is essential for learning-based anomaly detection techniques, which distinguish between normal and abnormal log sequences by using the log parsing results (i.e., templates) as learning features. However, despite the prevalent use of log parsing in anomaly detection, the importance of distinguishability has been surprisingly ignored in the log analysis community. This paper aims to highlight the significance of distinguishability in log parsing for anomaly detection. Furthermore, this is the first work to empirically demonstrate the importance of distinguishability after the theoretical framework proposed by Shin et al. (2021).

Though our objective here is not to identify the “best” log parsing and anomaly detection techniques, through our experiments, we found that there is no single best technique that significantly outperforms the others in all cases. In the future, to develop better log parsing techniques targeting anomaly detection, it would beneficial to focus on distinguishability, which has not been the case so far.

**Table 8:** Comparison with related empirical studies

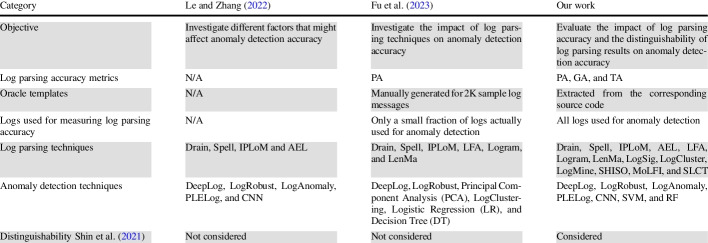	

## Related Work

Although individual techniques for log parsing and anomaly detection have been studied for a long time, systematic studies covering several techniques have only recently begun to emerge. For example, the most comprehensive evaluation studies on many log parsing techniques (Zhu et al. 2019; Dai et al. 2020; Khan et al. 2022) were conducted over the last four years. Similarly, the relationship between log parsing and anomaly detection has received little attention until very recently. Below, we summarize the recent studies related to this topic.

Shin et al. (2021) presented the first theoretical study considering the relationship between log parsing and anomaly detection. As described in Section 2.4, they established the concept of ideal log parsing results for anomaly detection. We adopted their theoretical foundation, especially the notion of *distinguishability* in log parsing results, and empirically showed that distinguishability is indeed essential for anomaly detection. To the best of our knowledge, our work is the first empirical study showing the importance of log parsing distinguishability for anomaly detection.

As explained in Section 3, Le and Zhang (2022) presented an empirical study on factors that could affect anomaly detection accuracy. Although a part of their study investigated the impact of log parsing on anomaly detection accuracy, they investigated four log parsing techniques but did not assess the impact of log parsing accuracy. As a result, they only showed that using different log parsing techniques leads to different anomaly detection accuracy scores. In our study, on the other hand, we explicitly measured log parsing accuracy, collected 160 pairs of log parsing accuracy and anomaly detection accuracy values using different combinations of log parsing and anomaly detection techniques, and showed that there is no strong correlation between log parsing accuracy and anomaly detection accuracy.

During the writing of this paper, Fu et al. (2023) also presented an empirical study on the impact of log parsing on anomaly detection performance. Although their motivation and research questions are close to ours, there are several key differences. First, for measuring log parsing accuracy, they used the manually generated, error-prone oracle templates (Khan et al. 2022) provided with the 2K log messages randomly sampled by Zhu et al. (2019). In other words, only a very small fraction of the logs used for anomaly detection was used to measure log parsing accuracy in their study. In our study, however, the same logs used for anomaly detection are used to measure log parsing accuracy, and the oracle templates are directly extracted from the corresponding source code. Second, they considered only one log parsing accuracy metric (GA), whereas we considered all three log parsing metrics (GA, PA, and TA) since different metrics assess complementary aspects of log parsing (Khan et al. 2022). Third, log parsing distinguishability, which is an essential factor that substantially affects anomaly detection accuracy (as shown in our RQ2), is only considered in our study. Finally, they only considered two deep learning-based anomaly detection techniques (DeepLog and LogRobust), and focused also on more traditional machine learning approaches (such as Principal Component Analysis, clustering, logistic regression, and decision trees). Such differences allow us to report new findings and provide concrete recommendations, as summarized in Section 6.

Wu et al. (2023) recently presented an empirical study on the effectiveness of log representation for machine learning-based anomaly detection. They considered different log representation techniques, such as FastText (Joulin et al. 2016), Word2Vec (Mikolov et al. 2013), TF-IDF (Salton and Buckley 1988) and BERT (Devlin et al. 2018), used to convert textual log data into numerical feature vectors for machine learning algorithms, such as Support Vector Machine, Logistic Regression, Random Forest, CNN, and LSTM. As a part of their study, they investigated the impact of log parsing on anomaly detection when used with different log representation techniques (in particular, FastText and Word2Vec). The empirical results showed that, in general, using log parsing (i.e., Drain (He et al. 2017)) improves the quality of log representations (over raw, unparsed data) and thereby the performance of anomaly detection; they also reported that some models (e.g., CNN and LSTM) are less sensitive to whether the log data is parsed or not, possibly due to the strong feature extraction and representation ability, and can offset the impact of noise generated by log parsing. In addition to these results, they also investigated the impact of additionally refining log parsing results using regular expressions and the impact of using different log parsing techniques. The results showed that refining log parsing results do not significantly increase anomaly detection performance but using different log parsing techniques yields slight variations in anomaly detection performance. However, for these additional investigations, they used only one anomaly detection technique (i.e., Logistic Regression) and two log parsing techniques (i.e., Drain (He et al. 2017) and LogPPT (Le and Zhang 2023)). Furthermore, they did not study the relationship between log parsing accuracy and anomaly detection accuracy. On the contrary, we use 13 log parsing techniques and 5 DL-based anomaly detection techniques to comprehensively investigate the relationship between log parsing accuracy and anomaly detection accuracy.

Table 8 summarizes the key differences between the closely-related previous empirical studies (i.e., Le and Zhang (2022); Fu et al. (2023)) and our work.

## Conclusion and Future Work

In this paper, we reported on a comprehensive empirical study investigating the impact of log parsing on anomaly detection accuracy, using 13 log parsing techniques, five DL-based and two ML-based anomaly detection techniques on three publicly available log datasets. When analyzing log parsing results for anomaly detection, we were surprised not to find any significant relationship between log parsing accuracy and anomaly detection accuracy, regardless of metric used for the former (including GA, PA, and FTA). This implies that, as opposed to common research practice to date, we can no longer select a log parsing technique purely based on its accuracy when used for anomaly detection. Instead, we experimentally confirmed existing theoretical results showing that the distinguishability of log parsing results plays an essential role in achieving accurate anomaly detection. It is therefore highly recommended to consider distinguishability when utilizing log parsing results as input for anomaly detection.

As part of future work, we plan to extend our study with more publicly available datasets and log parsing techniques (Le and Zhang 2023; Tao et al. 2023), which were published during the writing of this paper, to increase the generalizability of our results. We also aim to include state-of-the-art few-shot anomaly detection techniques (Huang et al. 2022; Pang et al. 2021), which require only a limited amount of training data and could be more effective in practice. We also plan to provide a more granular analysis of distinguishability for log parsing results by defining a new metric that assesses the degree of distinguishability. Finally, we plan to assess the performance of anomaly detection techniques that do not require log parsing (Le and Zhang 2021; Mvula et al. 2023; Nedelkoski et al. 2020).

## Data Availability

The replication package of our empirical evaluation (including the Python implementations for log parsing techniques, anomaly detection techniques, helper scripts, and datasets) is available on Figshare Khan et al. (2024).

## Footnotes

1 Note that a log is different from a log file. In practice, *one* log file may contain *many* logs representing the execution flows of different components/sessions. For example, an HDFS (Hadoop Distributed File System) log file contains many logs, distinguished by file block IDs, each representing an independent execution for a specific block.

2 We used the implementations from the scikit-learn library Pedregosa et al. (2011).
